# Hypoxemia-induced Wellens-like Electrocardiogram Pattern in Acute Exacerbation of Chronic Obstructive Pulmonary Disease

**DOI:** 10.31662/jmaj.2025-0056

**Published:** 2025-06-13

**Authors:** Hirohito Sano, Yuri Yamamoto, Hisatoshi Sugiura

**Affiliations:** 1 Department of Respiratory Medicine, Tohoku University Graduate School of Medicine, Sendai, Japan; 2 Department of Respiratory Medicine, South Miyagi Medical Center, Sendai, Japan

**Keywords:** Wellens syndrome, Wellens like ECG change, hypoxemia, chronic obstructive pulmonary disease

A 69-year-old male with severe coronary artery disease and proximal left anterior descending (LAD) artery stenosis presented with an acute exacerbation of chronic obstructive pulmonary disease. Respiratory failure with PaO_2_ of 55 mmHg was noted, and computed tomography showed extensive emphysema and bronchial wall thickening (Figure 1A). Electrocardiography (ECG) revealed Wellens-like changes with biphasic T waves in V2-V3 (Figure 1B). Treatment with ceftriaxone, systemic glucocorticoids, and bronchodilators improved hypoxemia, followed by the resolution of the ECG changes (Figure 1C and D).

**Figure 1.**
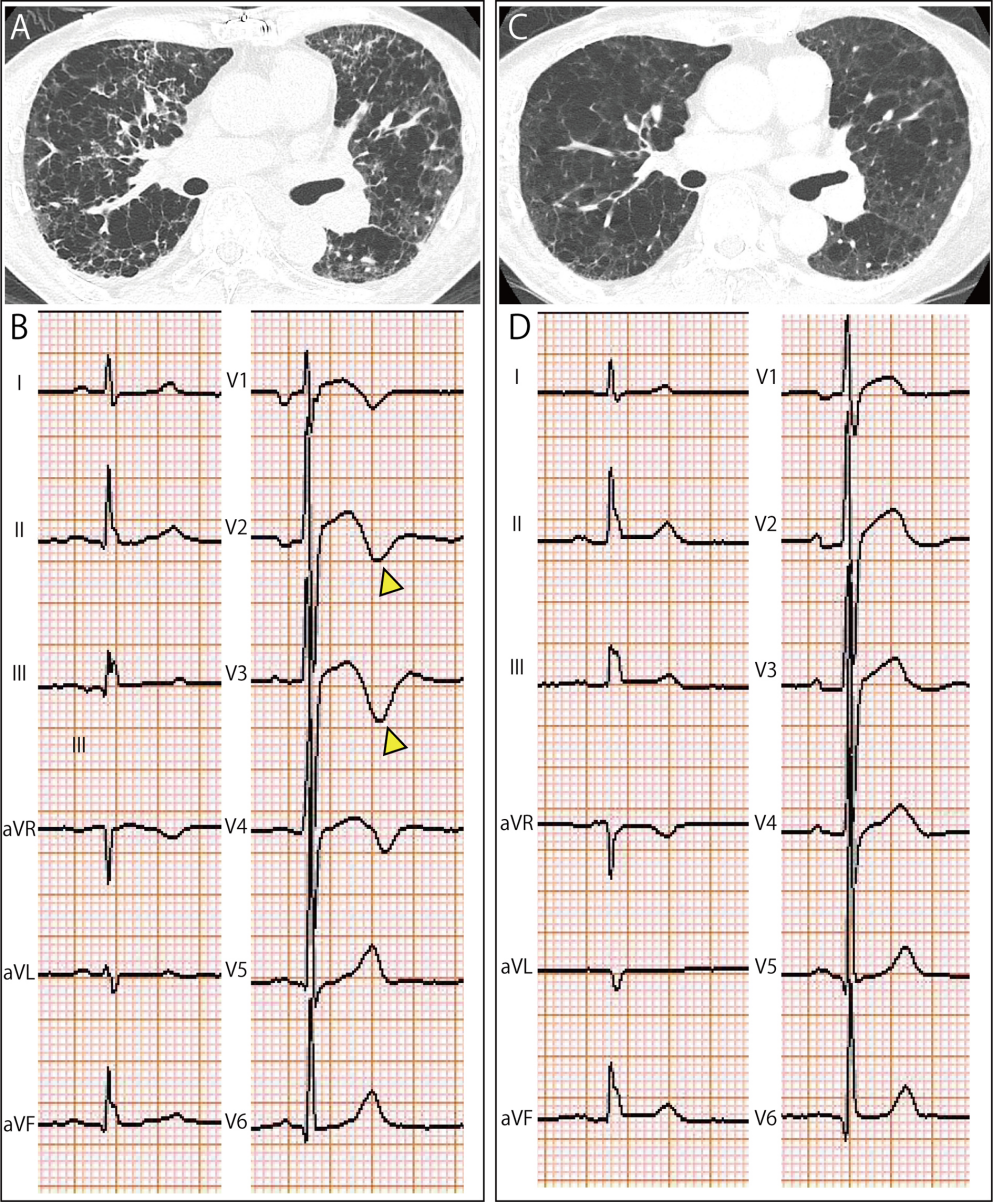
(A) Initial axial chest computed tomography revealing diffuse emphysematous changes and circumferential bronchial wall thickening. (B) Initial 12-lead electrocardiogram showing biphasic T waves in precordial leads V2-V3 (yellow arrowheads). (C) Follow-up axial chest computed tomography nine days after treatment initiation demonstrating reduced bronchial wall thickening. (D) Follow-up 12-lead electrocardiogram showing resolution of T wave abnormalities in precordial leads V2-V3.

Wellens syndrome, characterized by specific T-wave morphology in precordial leads, traditionally indicates critical LAD stenosis and a risk of anterior wall myocardial infarction (MI) ^(1)^. This case demonstrates that severe hypoxemia can induce Wellens-like ECG changes. Right ventricular overload from pulmonary embolism can present as T-wave inversion in precordial leads, requiring differentiation from Wellens-like ECG changes. However, echocardiography in this case did not reveal right ventricular strain. The observed Wellens-like ECG changes in this patient were likely attributable to multiple factors. Beyond the primary insult of oxygen supply-demand mismatch induced by hypoxemia, potential contributing mechanisms include myocardial stunning, microvascular dysfunction, and systemic inflammation ^(2)^. This pattern of oxygen supply-demand mismatch closely aligns with the pathophysiology of type 2 MI. According to the Fourth Universal Definition of MI, type 2 MI is defined as myocardial injury due to an imbalance between oxygen supply and demand ^(3)^. We observed a slight elevation in high-sensitivity cardiac troponin I (47.7 pg/mL; threshold: 26.2 pg/mL), suggesting myocyte injury. However, the magnitude of troponin elevation was substantially lower than that typically encountered in acute MI, indicating that complete or prolonged ischemia was unlikely to be the primary driver of myocardial compromise in this case. This observation suggests that Wellens-like ECG changes may occur in the context of severe hypoxemia, warranting further study.

## Article Information

### Conflicts of Interest

None

### Author Contributions

Hirohito Sano conceived and designed the experiments; Hirohito Sano and Yuri Yamamoto contributed to the interpretation of data; Hirohito Sano and Hisatoshi Sugiura approved the final version to be submitted.

### Informed Consent

Written informed consent was obtained from the patient for the publication of this case report and any accompanying images.

### Patient Consent

Consent to publish the details of the case was obtained from the patient.
